# Predicting Tumor Volume in Primary Hyperparathyroidism From Preoperative Clinical Data

**DOI:** 10.1210/clinem/dgae185

**Published:** 2024-03-25

**Authors:** Tomoyoshi Nakai, Kiyomi Horiuchi, Takahiro Okamoto

**Affiliations:** Department of Endocrine Surgery, Tokyo Women’s Medical University, Shinjuku-ku, Tokyo 162-8666, Japan; Department of Endocrine Surgery, Tokyo Women’s Medical University, Shinjuku-ku, Tokyo 162-8666, Japan; Department of Endocrine Surgery, Tokyo Women’s Medical University, Shinjuku-ku, Tokyo 162-8666, Japan

**Keywords:** primary hyperparathyroidism, parathyroidectomy, parathyroid hormone, calcium, parathyroid adenoma

## Abstract

**Context:**

Primary hyperparathyroidism (PHPT) is an endocrine disorder that is treated surgically, and some correlation between the size of the responsible lesion and preoperative clinical data is assumed.

**Objective:**

The purpose of this study was to predict tumor volume of the lesion responsible for PHPT from preoperative clinical data.

**Methods:**

Participants comprised patients with surgically treated PHPT who underwent initial surgery in our department between January 2000 and December 2021. The volume of parathyroid gland removed was used as the primary outcome and associations with preoperative clinical data were assessed by multivariable analysis.

**Results:**

A positive correlation was identified between parathyroid tumor volume and both preoperative intact parathyroid hormone (PTH) (Spearman's r = 0.503) and calcium values (Spearman's r = 0.338). Data for intact PTH value and tumor volume were logarithmically transformed (ln-PTH = log-transformed intact PTH value; ln-volume = log-transformed tumor volume). Multiple regression analysis revealed male sex, ln-PTH and calcium values as significant predictors of ln-volume, with standardized regression coefficients of 0.213 (95% CI 0.103-0.323), 0.5018 (95% CI 0.4442-0559), and 0.322 (95% CI 0.0339-0.149), respectively. The adjusted R^2^ for this model is 0.320.

**Conclusion:**

Preoperative serum intact PTH value is associated with tumor volume of the lesion responsible for PHPT. A rough estimation of the tumor size would provide responsible physicians with opportunities to add further imaging tests or plan appropriate surgical strategies.

Primary hyperparathyroidism (PHPT) is a surgical endocrine disorder. Guidelines for surgical intervention and management guidelines have been established in many countries, and parathyroidectomy is considered the only definitive treatment (1, 2). Since PHPT is most often (80-85%) due to a single adenoma, targeted selective or focused approaches gained support when Russell and Tibblin pioneered these surgeries in the early 1980s (3, 4). In the 1990s, Irvin devised a method of rapid intraoperative PTH monitoring, allowing functional decisions to be made according to the so-called Miami criteria, and the focused approach became widespread in addition to the traditional bilateral searching approach (5, 6). On the other hand, bilateral exploration is recommended for patients with unknown localization or known or suspected multigland disease (7). If the location of the lesion responsible for primary hyperparathyroidism is identified preoperatively, the focused approach can achieve disease cure in ≥95% of cases, a rate almost as high as the traditional bilateral searching approach (2, 8). Patients with at least 1 preoperative localizing study can undergo the focused approach and anticipate a cure rate of 99% (9). Models have also been developed that distinguish between a single-gland and multigland parathyroid disease in PHPT (10, 11). Preoperative diagnostic imaging techniques such as ultrasonography, computed tomography, and ^99m^Tc-methoxy-isobutyl-isonitrile (MIBI) scintigraphy are used to localize the gland, but preoperative localization may be incorrect or surgical treatment may prove unsuccessful (12, 13). Preoperative prediction of the size and localization of the responsible lesion would be very useful to the surgeon, helping to determine the extent of the search and the approach to be adopted.

Some correlations presumably exist between the size of the responsible lesion and preoperative clinical data, such as serum intact PTH and calcium values. At least 7 investigations have reported on the correlations between preoperative serum intact PTH or calcium values in patients with PHPT and the volume or weight of the responsible parathyroid tumor (14-20). Five of those reports sought to correlate preoperative biochemical data with the weight of the parathyroid tumor (14-16, 18, 19), with 4 identifying a correlation (14, 16, 18, 19). However, the weight of the resected specimen obviously cannot be determined prior to the surgery. A more clinically realistic option is to develop a predictive model for the volume of the responsible parathyroid tumor using preoperative biochemical data. Although some reports have stated that preoperative intact PTH value may be useful in predicting parathyroid tumor volume (16-18), the interpretation of research findings among researchers has been quite inconsistent, particularly between those few large studies with ≥500 cases. Furthermore, postoperative biochemical cure of the target population has not been confirmed in some studies (14, 16, 20). Here, we conducted a large, retrospective study with 1086 participants to predict the volume of the lesion responsible for PHPT from preoperative clinical data.

## Materials and Methods

### Design and Patients

We retrospectively reviewed and analyzed the computerized medical records of all patients diagnosed with PHPT who underwent initial surgery between January 2000 and December 2021 at our institution. Patients with multigland diseases (such as multiple endocrine neoplasia, familial hypocalciuric hypercalcemia, metastases of parathyroid carcinoma, recurrent or persistent hyperparathyroidism) and patients deemed unsuitable as study subjects were excluded. In all patients, the pathological diagnosis confirmed parathyroid adenoma, atypical parathyroid adenoma, or parathyroid carcinoma.

### Data Collection

We manually reviewed the electronic medical charts and obtained age, sex, preoperative serum intact PTH, calcium, phosphorus, creatinine, and alkaline phosphatase values; operative reports and pathology reports were reviewed. Serum calcium values were corrected based on albumin values. If the serum albumin value was <4.0 g/dL, the serum calcium value was calculated as the measured serum calcium value (mg/dL) + (4.0 − serum albumin values [g/dL]). In our department, the serum alkaline phosphatase value was measured from the Japan Society of Clinical Chemistry (JSCC) transferable method, using a commercial kit from Roche Diagnostics KK, to the International Federation of Clinical Chemistry (IFCC) method, using a commercial kit from the Shino-Test Corporation in April 2021. The regression formulas for alkaline phosphatase value using the conventional JSCC (x) and IFCC (y) methods are y = 0.337x + 2.959 (21). The reference value was changed from 115-359 IU/mL to 38-113 IU/mL. It was decided to unify the reference value with the IFCC method. Biochemical data closest to the date of surgery within 7 days before surgery and obtained from the same blood draw were used. Although clinicians are likely to attempt to predict the size of the parathyroid tumor in outpatient settings, collection of biochemical data as close as possible (ie, during surgery) to the time at which the actual volume of the parathyroid tumor becomes known is considered appropriate, as surgical waiting periods differed. The diagnosis of hyperparathyroidism was based on elevated serum intact PTH and calcium values. Postoperative serum intact PTH and calcium values were checked, biochemical cure was determined with normalization of intact PTH and calcium values, and recurrent or persistent hyperparathyroidism was excluded. All patients underwent surgery under general anesthesia. The parathyroid gland, along with the surrounding fatty tissue, was removed without damaging the capsule. The 3 diameters (longitudinal, transversal, and sagittal) were measured for all removed parathyroid glands. To calculate gland volume, the parathyroid gland was considered as an ellipsoid (volume [mm^3^] = π/6 × a × b × c, where a, b, and c represent the 3 dimensions of the parathyroid gland).

### Study Outcomes

The primary outcome of this study was to predict parathyroid tumor volume from preoperative biochemical data. The secondary outcome was to determine the relationships between the parathyroid tumor volume and clinical characteristics such as age, sex, preoperative serum intact PTH, calcium, phosphorus, creatinine, and alkaline phosphatase values.

### Statistical Analysis

We summarized the patient characteristics using descriptive statistics. Categorical variables are described as counts and percentages. As continuous variables were tested using the Shapiro–Wilk method and did not show normal distributions, these data are provided as median, interquartile range. To compare parathyroid tumor volumes and other variables, the Wilcoxon rank-sum test was used, since parathyroid tumor volume did not show a normal distribution. To explore relationships between parathyroid tumor volume and other variables, including serum intact PTH value, we employed correlation analyses using Spearman's r, as normality could not be ensured in the distribution of preoperative biochemical data. Serum intact PTH, creatinine, alkaline phosphatase values and parathyroid tumor volumes were logarithmically transformed to approach normal distributions for multiple regression modeling (ln-PTH = log-transformed intact PTH value; ln-creatinine = log-transformed creatinine value; ln-alkaline phosphatase = log-transformed alkaline phosphatase value; ln-volume = log-transformed tumor volume). Multiple regression modeling with parathyroid tumor volume as a dependent variable was used to examine relationships between volume of the lesion responsible for PHPT and preoperative clinical data. An iterative approach was adopted to select appropriate covariates to maximize adjusted R^2^ values or to minimize the Akaike's information criterion for models. All statistical analyses were conducted in JMP Pro version 17.0.0 (SAS Institute Inc., Cary, NC, USA). We set a 2-sided *P* < .05 as indicative of statistical significance for type I error.

## Results

**Figure 1.**
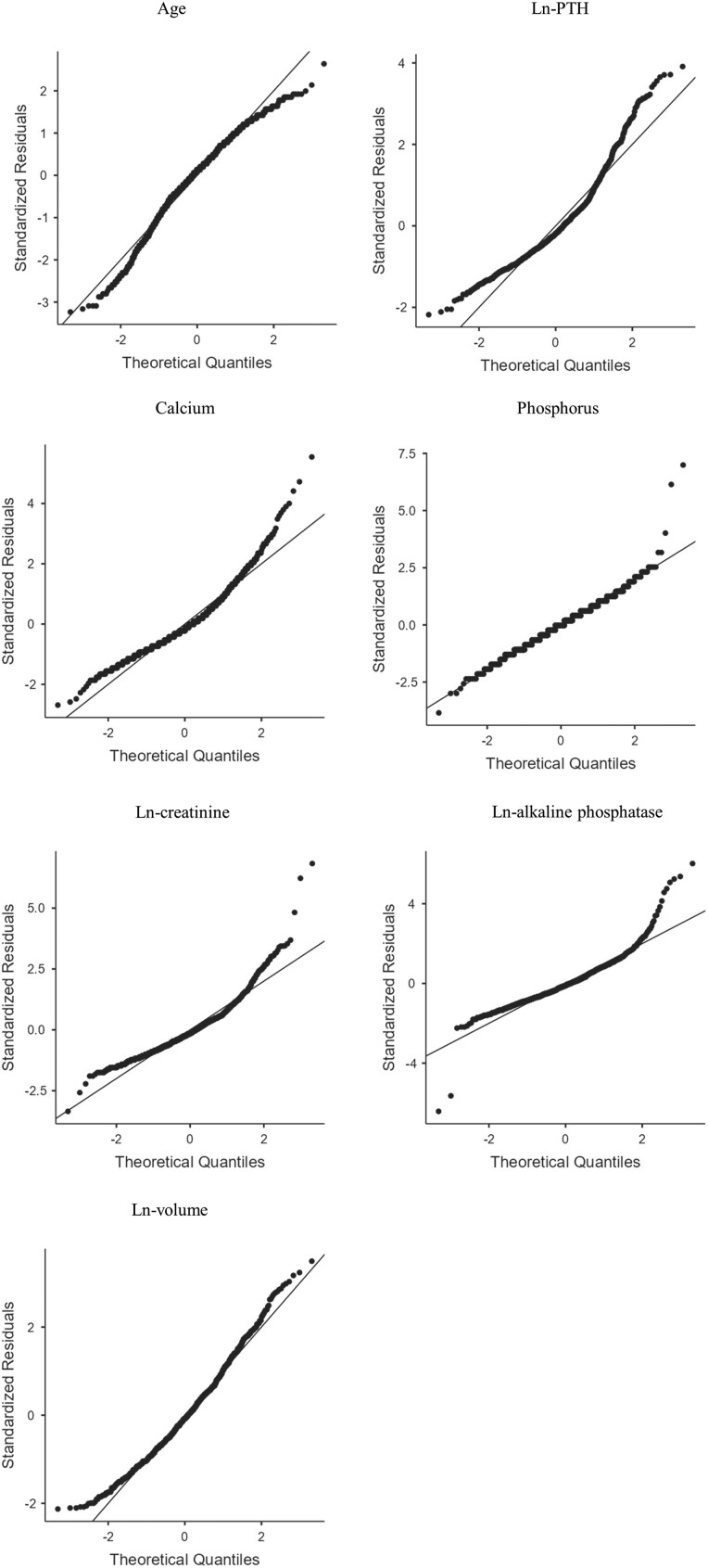
Deviation of the united line from the dotted line indicates non-normal distribution.

**Figure 2.**
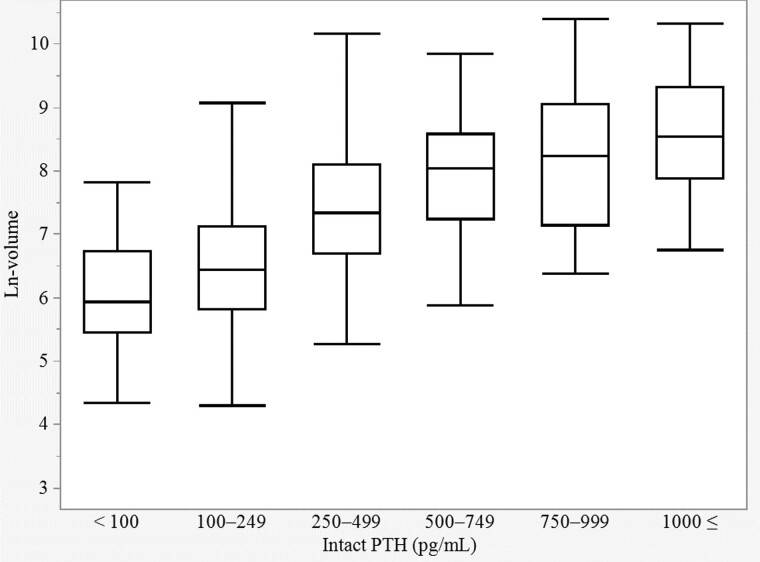
A box and whisker diagram was created using ln-volume as the vertical axis and classified by intact PTH.

**Figure 3.**
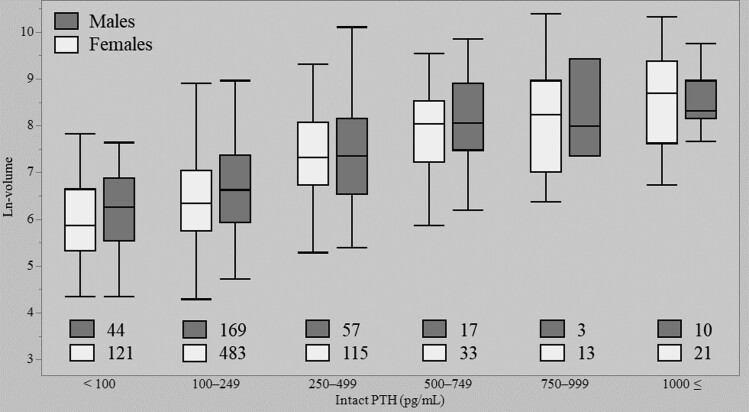
A box and whisker diagram was created using ln-volume as the vertical axis and classified by intact PTH and sex.

A total of 1086 patients were included in the study and further analyzed. Table 1 shows the clinical characteristics of study participants. Median age was 60 years (range 13-95 years) and 72.4% were females. Median values for preoperative serum intact PTH, calcium, phosphorus, creatinine, and alkaline phosphatase values and excised tumor volume were 163 pg/mL (range 117-247 pg/mL), 10.8 mg/dL (range 10.4-11.5 mg/dL), 2.5 mg/dL (range 2.2-2.8 mg/dL), 0.69 mg/dL (range 0.58-0.86 mg/dL), 106 U/L (range 80-149 U/L), and 754 mm^3^ (range 364-1606 mm^3^), respectively. A significant difference in parathyroid tumor volume was evident between men and women (*P* < .05; Table 2). Spearman's correlation coefficients were calculated for each pair of dependent and explanatory variables (Table 3). Spearman's correlation coefficients revealed parathyroid tumor volume was significantly associated with preoperative serum intact PTH, calcium, phosphorus, creatinine, and alkaline phosphatase values. In particular, parathyroid tumor volume showed a moderate correlation with intact PTH (r = 0.503, *P* < .05) and calcium value (r = 0.338, *P* < .05). Figure 1 presents q-q plots providing visual comparisons of sample quantiles to the corresponding theoretical quantiles for preoperative clinical data. Multiple regression analysis of factors potentially associated with parathyroid tumor volume revealed standardized regression coefficients of 0.213 (95% CI 0.103-0.323), 0.5018 (95% CI 0.4442-0.559), and 0.0915 (95% CI 0.0339-0.149) for male sex, ln-PTH, and calcium values, respectively (Table 4). This model showed fit measures of R^2^ = 0.322 and adjusted R^2^ = 0.320 (*P* < .001). In other words, approximately 30% of the variance in parathyroid tumor volume was explained by sex, serum intact PTH, and calcium values. Parathyroid tumor volume was significantly larger in males, with higher preoperative serum intact PTH and higher calcium values. As the correlation between preoperative serum intact PTH value and parathyroid tumor volume was strongest, a box and whisker diagram was created using ln-volume as the vertical axis and classified by intact PTH (Fig. 2). Median ln-volumes were 5.93 (IQR 5.44-6.73), 6.44 (IQR 5.82-7.12), 7.34 (IQR 6.70-8.08), 8.05 (IQR 7.27-8.54), 8.24 (IQR 7.29-8.86), and 8.55 (IQR 7.90-9.20) for intact PTH, for <100 pg/mL, between 100 and 249 pg/mL, 250 and 499 pg/mL, 500 and 749 pg/mL, 750 and 999 pg/mL, and >1000 pg/mL, respectively. From that graph, a rough indication of tumor volume was observed when intact PTH was within a certain range. Similarly, a box and whisker diagram classified by intact PTH and sex was also created (Fig. 3).

**Table 1.** Patient and disease characteristics

Characteristics	Median (interquartile range) or n (%)
Age (years)	60 (17)
Sex	
Female	786 (72.4%)
Male	300 (26.4%)
Intact parathyroid hormone (reference: 16-65 pg/mL)	163 (130)
Calcium (reference: 8.5-9.9 mg/dL)	10.8 (1.1)
Phosphorus (reference: 2.5-4.3 mg/dL)	2.5 (0.6)
Creatinine (reference: 0.48-0.79 mg/dL)	0.69 (0.28)
Alkaline phosphatase (reference: 38-113 U/L)	106 (69)
Parathyroid tumor volume (mm^3^)	754 (1243)

**Table 2.** Association of serum intact PTH, calcium values, and parathyroid tumor volume with sex

	Overall (n = 1086)	Male (n = 300)	Female (n = 786)	*P* value
Intact parathyroid hormone (pg/mL)*^a^*	163 (117-247)	168 (118-266)	160 (117-243)	.247
Calcium (mg/dL)*^a^*	10.8 (10.4-11.5)	11.0 (10.4-11.6)	10.8 (10.4-11.5)	.105
Parathyroid tumor volume (mm^3^)*^a^*	754 (364-1606)	904 (448-2159)	705 (339-1473)	<.05

^*a*^Median (25th-75th percentile).

**Table 3.** Spearman's correlation coefficient matrix

	Age	Intact PTH	Calcium	Phosphorus	Creatinine	Alkaline phosphatase
Intact PTH	0.064*	—				
Calcium	0.027	0.512*	—			
Phosphorus	−0.098	−0.432*	−0.333*	—		
Creatinine	0.173*	0.101*	0.220*	−0.165*	—	
Alkaline phosphatase	−0.091*	0.393*	0.351*	−0.054*	−0.091	—
Volume	0.021	0.503*	0.338*	−0.267*	0.123*	0.243*

Abbreviation: PTH, parathyroid hormone.

^*^ *P* < 0.05.

**Table 4.** Multiple regression model to predict ln-volume

Explanatory variable	Standardized coefficient (95% CI)	*P* value
Sex: Male vs Female	0.213 (0.103-0.323)	<.001
ln-parathyroid hormone	0.5018 (0.4442-0.559)	<.001
Calcium	0.0915 (0.0339-0.149)	.002
Constant	0.000	<.001

## Discussion

This is the largest study to attempt prediction of parathyroid tumor volume from preoperative biochemical data in the English-language literature and included 1086 patients with sporadic primary hyperparathyroidism. The results showed that parathyroid tumor volume was moderately correlated with preoperative serum intact PTH and calcium levels. In clarifying the relationship between preoperative biochemical data and parathyroid tumor volume, multiple regression analysis showed that parathyroid volume was significantly larger in males, with higher preoperative serum PTH and higher calcium levels.

A few investigators have attempted to predict parathyroid tumor size from clinical data in PHPT. They found that the strength of associations between the size and the preoperative serum levels of intact PTH or calcium ranged from 0.08 (15) to 0.51 (14, 17), leading to inconsistent conclusions on predictability. The variability of the correlation coefficient might stem from several reasons, as follows. First, the timing of blood sample collection varied among the studies. Some used the most recent values obtained before surgery (14, 15, 18-20), while others did not describe the details. Second, the weight or volume of the resected parathyroid tumor was measured in different ways. The weight scales used were not reproducible, and although all studies have considered the parathyroid tumor as an ellipsoid for calculations of volume (15-18, 20), reproducibility has not been demonstrated. Third, parathyroid tumors causing PHPT may comprise chief cells, oxyphil cells, or a mixed-cell composition. Chief cells are thought to be the primary secretory cells in the parathyroid glands. The proportion of chief and oxyphil cells occupying parathyroid tumors impacts the biochemical data. However, no studies have examined the pathological picture and confirmed in detail the types of cells occupying parathyroid tumors. Fourth, all studies calculated correlation coefficients as an index of correlation, but not all stated whether Pearson's or Spearman's correlation coefficient was used (15, 18). Studies stating that Pearson's correlation coefficient was used did not confirm the normality of continuous variables (17, 20). Finally, the study with the largest sample size lacked descriptions of the biochemical cure for the disease and histopathological findings.

A unique aspect of our study was that a box and whisker diagram was created with ln-volume on the vertical axis and classified by intact PTH, as the correlation between ln-PTH and ln-volume was the strongest. Parathyroid tumor volume was thus seen to increase as intact PTH increases. That graph allowed a rough indication of the range of the parathyroid tumor volume. For instance, suppose a patient with a preoperative intact PTH value of 800 pg/mL whose ultrasonography suggests a parathyroid tumor with a volume of 500 mm^3^ log-transformed to 6.21. Referring to Fig. 2, we could proceed with further imaging studies or extensive neck exploration, considering the possibility of a responsible lesion elsewhere.

The present study showed some limitations. The investigation was retrospective in design and the timing of preoperative blood tests could not be standardized to the outpatient setting, so blood tests were used on admission. This may be interpreted differently than when parathyroid tumors are assessed in the outpatient setting. Serum intact PTH level may also be elevated due to vitamin D deficiency, possibly secondary to hyperparathyroidism (22, 23). As measurement of serum 25(OH)D values has only become available in recent years, our study did not collect vitamin D values. The extent of mixed chief and oxyphil cells and the extent to which these cell populations influenced the pathology are unknown.

In conclusion, our study is the largest study to show relationships between preoperative biochemical data and the responsible lesion in primary hyperparathyroidism due to sporadic and single-adenoma disease. Preoperative serum intact PTH values correlate with tumor volume in functional parathyroid tumors, but the utility of intact PTH values appears limited for accurately predicting tumor size preoperatively. However, if the preoperative intact PTH is extremely high and preoperative imaging modalities such as ultrasonography, computed tomography, or MIBI scintigraphy suggest that the responsible lesion is correspondingly small, further imaging should be undertaken, not only focusing on the preoperatively determined responsible lesion, but also using unilateral or bilateral approaches. Furthermore, functional confirmation of the decrease in serum intact PTH value during surgery would maximize the chance of cure.

### Conclusions

Preoperative serum intact PTH values are associated with tumor volume of the responsible lesion in primary hyperparathyroidism. The ability of this information to accurately predict tumor volume appears limited, but rough estimation appears feasible.

## Data Availability

Some or all datasets generated during and/or analyzed during the current study are not publicly available but are available from the corresponding author on reasonable request.

## Abbreviations

IFCC: International Federation of Clinical Chemistry
JSCC: Japan Society of Clinical Chemistry
MIBI: ^99m^Tc-methoxy-isobutyl-isonitrile
PHPT: primary hyperparathyroidism
PTH: parathyroid hormone
